# Supplementary material for: Comparison of patient-reported outcomes at one year after injury between limb salvage and amputation: A prospective cohort study
Source: PLoS One. 2022 Sep 19;17(9):e0274786. doi: 10.1371/journal.pone.0274786 (PMC9484632; doi:10.1371/journal.pone.0274786)
Supplement: Attachment — Submitted filename: 22.06.08Response_to_Reviewers.docx [file pone.0274786.s001.docx]

June 8, 2022

Emily Chenette

Editor-in-Chief

*PLOS ONE*

Dear Editor:

We/I wish to re-submit the manuscript titled “**Comparison of patient-reported outcomes at one year after injury between limb salvage and amputation: A prospective cohort study** .” The manuscript ID is D-22-06485.

We thank you and the reviewers for your thoughtful suggestions and insights. The manuscript has benefited from these insightful suggestions. I look forward to working with you and the reviewers to move this manuscript closer to publication in the *PLOS ONE*.

The manuscript has been rechecked and the necessary changes have been made in accordance with the reviewers’ suggestions. The responses to all comments have been prepared and given below. Changes in the manuscript have been highlighted in yellow.

Thank you for your consideration. I look forward to hearing from you.

Sincerely,

Taketo Kurozumi

Trauma and Reconstruction Center, Teikyo University Hospital

2-11-1, Kaga, Itabashi, Tokyo, 173-8606, Japan

Telephone: +81-3-3964-1211

Fax number: +81-3-5375-0854

E-mail address: [taketo_kurozumi@m6.dion.ne.jp](mailto:taketo_kurozumi@m6.dion.ne.jp)

Response to Reviewers

Journal Requirements:

When submitting your revision, we need you to address these additional requirements.

1. Please ensure that your manuscript meets PLOS ONE's style requirements, including those for file naming. The PLOS ONE style templates can be found at

<https://journals.plos.org/plosone/s/file?id=wjVg/PLOSOne_formatting_sample_main_body.pdf>  and

<https://journals.plos.org/plosone/s/file?id=ba62/PLOSOne_formatting_sample_title_authors_affiliations.pdf>

Response: Thank you for the information. We have made the required changes.

2. In your Data Availability statement, you have not specified where the minimal data set underlying the results described in your manuscript can be found. PLOS defines a study's minimal data set as the underlying data used to reach the conclusions drawn in the manuscript and any additional data required to replicate the reported study findings in their entirety. All PLOS journals require that the minimal data set be made fully available. For more information about our data policy, please see <http://journals.plos.org/plosone/s/data-availability>.

Upon re-submitting your revised manuscript, please upload your study’s minimal underlying data set as either Supporting Information files or to a stable, public repository and include the relevant URLs, DOIs, or accession numbers within your revised cover letter. For a list of acceptable repositories, please see <http://journals.plos.org/plosone/s/data-availability#loc-recommended-repositories>. Any potentially identifying patient information must be fully anonymized.

Important: If there are ethical or legal restrictions to sharing your data publicly, please explain these restrictions in detail. Please see our guidelines for more information on what we consider unacceptable restrictions to publicly sharing data: <http://journals.plos.org/plosone/s/data-availability#loc-unacceptable-data-access-restrictions>. Note that it is not acceptable for the authors to be the sole named individuals responsible for ensuring data access.

We will update your Data Availability statement to reflect the information you provide in your cover letter.

Response: Thank you for the information. We have added the following to the title page.

Request can be made to the Japanese Society for Fracture Repair secretariat (1-8-6, Shintomi Chuoku Tokyo 104-0041 Japan) or to [jsfr@jsfr.jp](mailto:jsfr@jsfr.jp) and those who meet the criteria for access to anonymized patient level data will be granted data access.

3. Thank you for stating the following financial disclosure:

“No. The funders had no role in study design, data collection and analysis, decision to publish, or preparation of the manuscript.”

At this time, please address the following queries:

a)        Please clarify the sources of funding (financial or material support) for your study. List the grants or organizations that supported your study, including funding received from your institution.

b)        State what role the funders took in the study. If the funders had no role in your study, please state: “The funders had no role in study design, data collection and analysis, decision to publish, or preparation of the manuscript.”

c)        If any authors received a salary from any of your funders, please state which authors and which funders.

d)        If you did not receive any funding for this study, please state: “The authors received no specific funding for this work.”

Please include your amended statements within your cover letter; we will change the online submission form on your behalf.

Response: We have added the following to the cover letter and it has been added to the Title page.

The authors received no specific funding for this work.

Comments to the Author

5. Review Comments to the Author

Reviewer #1: Summary:
The authors present a small cohort study of patients with severe lower limb fractures, an uncommon but profoundly disabling injury. Deciding on the optimal treatment (salvage or amputation) for patients with these injuries is recognised as one of the most difficult decisions a surgeon can face. The results of this study advance understanding of patient centered outcomes following these injuries, and will contribute to the literature aiding decision-making between amputation and limb salvage.

Response: Thank you for understanding our arguments.

Major issues:
Introduction & Discussion:
1) The Lower Extremity Assessment Project (LEAP) was a multi-centre, prospective cohort study between 1994 and 1997 that investigated the topic of this paper, and followed patients up at 2 (Bosse, NEJM) and 7 (MacKenzie, JBJS) years. The results of the LEAP study are now probably dated, and differ from the results of this study - however, the authors should report the key background knowledge on this topic in the introduction, and discuss their findings in the content of this previous work. Indeed, the authors suggest in the introduction that "prospective studies based on patient reported outcomes are extremely rare", yet the LEAP study is just this, and has provided the largest body of knowledge on the topic.

Response: Thank you for this suggestion. We have added its content in the background and discussion. Page 3, Line 65-71; Page 14, Lines 288-290

Methods:
1) Consider using the Strobe reporting guideline for cohort studies

Response: Thank you for your suggestion. We have rewritten some of them to conform to the guidelines. Page 3, Lines 79, 81; Page 4, Lines 86, 89.

Minor issues:
Introduction:
1) The introduction is short, well written and highlights a clear rationale for the study and potential knowledge gap. However, there are few references. Please could the authors consider referencing more of the statements if possible.

Response: Thank you very much. We have added some additional references and background information there. Page 3, Lines 65, 66, 68, 71.

Results:
1) The magnitude of the functional disability in both groups is substantial, this in itself is an important finding, that is not highlighted. Could the authors consider showing this in graphical format (before - after LEFS scores)? And place in context what a LEFS score of 33 (average for amputees) means - on average moderate to quite a bit of difficulty with all activities of daily living!

Response: Thank you for this suggestion. We have added its content in the result and discussion. Pages 10-11, Lines 176-206; 217-218

Discussion:
1) Consider discussing the possible reasons for the considerable impact these injuries have on mental health ( a key finding of this paper)

Response: Thank you for this insightful comment. We have added a discussion around this topic to the discussion section. Page 13, Lines 275-278
