# Supplementary material for: Comparison of patient-reported outcomes at one year after injury between limb salvage and amputation: A prospective cohort study
Source: PLoS One. 2022 Sep 19;17(9):e0274786. doi: 10.1371/journal.pone.0274786 (PMC9484632; doi:10.1371/journal.pone.0274786)
Supplement: Attachment — Submitted filename: 22.07.03Response to Reviewers.docx [file pone.0274786.s002.docx]

July 3, 2022

Emily Chenette

Editor-in-Chief

*PLOS ONE*

Dear Editor:

We/I wish to re-submit the manuscript titled “**Comparison of patient-reported outcomes at one year after injury between limb salvage and amputation: A prospective cohort study** .” The manuscript ID is D-22-06485R1.

We thank you and the reviewers for your thoughtful suggestions and insights. The manuscript has benefited from these insightful suggestions. I look forward to working with you and the reviewers to move this manuscript closer to publication in the *PLOS ONE*.

The manuscript has been rechecked and the necessary changes have been made in accordance with the reviewers’ suggestions. The responses to all comments have been prepared and given below. Changes in the manuscript have been highlighted in yellow.

Thank you for your consideration. I look forward to hearing from you.

Sincerely,

Taketo Kurozumi

Trauma and Reconstruction Center, Teikyo University Hospital

2-11-1, Kaga, Itabashi, Tokyo, 173-8606, Japan

Telephone: +81-3-3964-1211

Fax number: +81-3-5375-0854

E-mail address: [taketo_kurozumi@m6.dion.ne.jp](mailto:taketo_kurozumi@m6.dion.ne.jp)

**6. Review Comments to the Author**

Reviewer #1: Minor issue:

Thank you for including reference to the LEAP study body of work.

This was a large multi-centre, prospective observational study, that took place between 1994 and 1997, and remains one of the most important civilian studies on the topic.

In the revised introduction, the sentence "Moreover, many previous investigations on this topic are retrospective cohort studies or meta-analyses on retrospective studies, such as the LEAP study, whereas prospective cohort studies based on patient-reported outcomes are extremely rare [1-3]." implies that the LEAP study is a retrospective cohort study or a meta-analysis of retrospective studies, which it is neither.

Please consider rewording to something like: "Moreover, many previous investigations on this topic are retrospective cohort studies or meta-analyses on retrospective studies, whereas prospective cohort studies based on patient-reported outcomes, such as the LEAP study, are extremely rare [1-3]."

Response: Thank you for this suggestion. We have made the required changes. Page 5, Line 68-69
